# Antagonistic maternal and direct effects of the leptin receptor gene on body weight in pigs

**DOI:** 10.1371/journal.pone.0246198

**Published:** 2021-01-28

**Authors:** Emma Solé, Roger Ros-Freixedes, Marc Tor, Josep Reixach, Ramona N. Pena, Joan Estany

**Affiliations:** 1 Department of Animal Science, University of Lleida-Agrotecnio Center, Lleida, Catalonia, Spain; 2 Selección Batallé S.A., Riudarenes, Catalonia, Spain; University of Bern, SWITZERLAND

## Abstract

Maternal effects on offspring growth can impact survival and evolution of natural and domesticated populations. Genetic correlation estimates often support a negative relationship between direct and maternal effects. However, the genetic underpinnings whereby this antagonism operates are unclear. In pigs, sow feeding status and body composition condition piglet development and growth. We hypothesized that variants in genes impacting these traits may be causative of maternal influences that could be antagonistic to the direct effects for piglet growth. A recessive missense mutation (C>T) in the porcine leptin receptor (*LEPR*) gene (rs709596309) has been identified as the possible causal polymorphism for increased feed intake and fatness. Using data from a Duroc line, we show that the TT sows exerted a negative impact on the body weight of their offspring at the end of the growing period of similar extent to the positive direct effect of the TT genotype over each individual. Thus, TT pigs from TT dams were about as heavy as CC and CT (C–) pigs from C–dams, but TT pigs from C–dams were around 5% heavier than C–pigs from TT dams. In contrast, body composition was only influenced by *LEPR* direct effects. This antagonism is due to a higher propensity of TT pigs for self-maintenance rather than for offspring investment. We show that TT pigs consumed more feed, favored fatty acid uptake over release, and produced lighter piglets at weaning than their C–counterparts. We conclude that *LEPR* underlies a transgenerational mechanism for energy distribution that allocates resources to the sow or the offspring according to whether selective pressure is exerted before or after weaning.

## Introduction

Maternal effects occur when a mother influences her offspring beyond the direct effects of the genetic material that she transmitted to them. The importance of maternal influence on growth has been long known in livestock production. As already noted by Varro (1^st^ century BCE) in his book on farming, a sow with piglets should be fed more bountifully because they will grow thin if she gives little milk [1]. Maternal effects on progeny growth can have a significant impact on offspring survival and therefore on the evolution of natural and domesticated populations [2]. Maternal effects can accelerate or impede the rate of response to selection of a trait, depending on whether their effect on the phenotype of the offspring is positive or negative [3]. Traits that result in maternal effects for growth traits, such as milk production, are themselves the result of the joint action of the maternal genotype and the environment, and thus they can also be subjected to genetic variation and selection. There are many examples of maternal environmental influences and how they can affect offspring development. One of the most well-known is precisely the favorable effect of the sow feeding status on piglet growth [4, 5]. On the other hand, results indicate rather consistently a negative relationship between direct and maternal genetic effects, particularly in pigs [6, 7]. However, the genetic underpinnings whereby this antagonism operates are unclear.

Feeding young piglets increases energy demands for females. During lactation, sows must simultaneously cope with self-maintenance and milk production, if not with their own growth [8]. Voluntary feed intake does not generally meet such surplus of energy demands [9, 10] and lactating sows have to resort to body reserves to support milk production. Since sow body weight and backfat thickness are indicators of the ability to mobilize body reserves [11], we hypothesize that variants in genes impacting these traits or body energy balance may be causative of maternal influences. Numerous experiments have been purposely designed to unravel the genetic architecture of body fat content and distribution in pigs, especially using F_2_ crosses between divergent populations [12]. There is evidence for quantitative trait loci for fatness on chromosome 6 that map close to the leptin receptor (*LEPR*) gene in Iberian [13], Asian [14] and Duroc [15] breeds. Leptin is a hormone predominantly secreted by white adipocytes that is known to regulate food intake and energy balance [16, 17]. Leptin deficiency causes excessive feed intake and energy savings and, consequently, greater body weight and fat mass. Defective leptin receptor expression produces similar obese phenotypes and hyperleptinemia, in an attempt to counteract the leptin resistance-like state triggered by the leptin receptor deficit [18]. A missense mutation (C>T) in exon 15 (exon 14 in former genome build versions) of the *LEPR* gene (rs709596309) has been identified as the possible causal polymorphism [13]. While fixed in Iberian, the T allele segregates in Duroc as full recessive, with TT pigs displaying increased serum leptin levels and overall fatness [19, 20]. Although there is less compelling evidence on the impact of this mutation on body weight, results from F_2_ crosses indicate that the T allele may also boost growth [21].

Functional mutations in the porcine *LEPR* gene can be a useful model to investigate the genetic basis of the interaction between direct and maternal genetic effects on individual development. Here, we address the role of maternal effects on body growth that stem from the *LEPR* rs709596309 variant using a Duroc line where the T allele was segregating at an intermediate frequency [20]. In F_2_ designs, direct and maternal effects are very difficult to disentangle due to no or very little variation in the genotype of the F_1_ dam, as well as to between-breed linkage disequilibrium. Single gene analysis within segregating populations overcomes these shortfalls. More specifically, we first show that the *LEPR* rs709596309 variant is causative of a maternal effect in pigs that is at odds with the direct effect for piglet growth. Then, we demonstrate that this antagonism is due to a bias of TT pigs for self-maintenance rather than offspring investment. Based on these findings, we finally discuss evolutionary insights into the role and significance of *LEPR* as a transgenerational mechanism for energy allocation.

## Results

### Direct and maternal effects on growth

**Fig 1:**
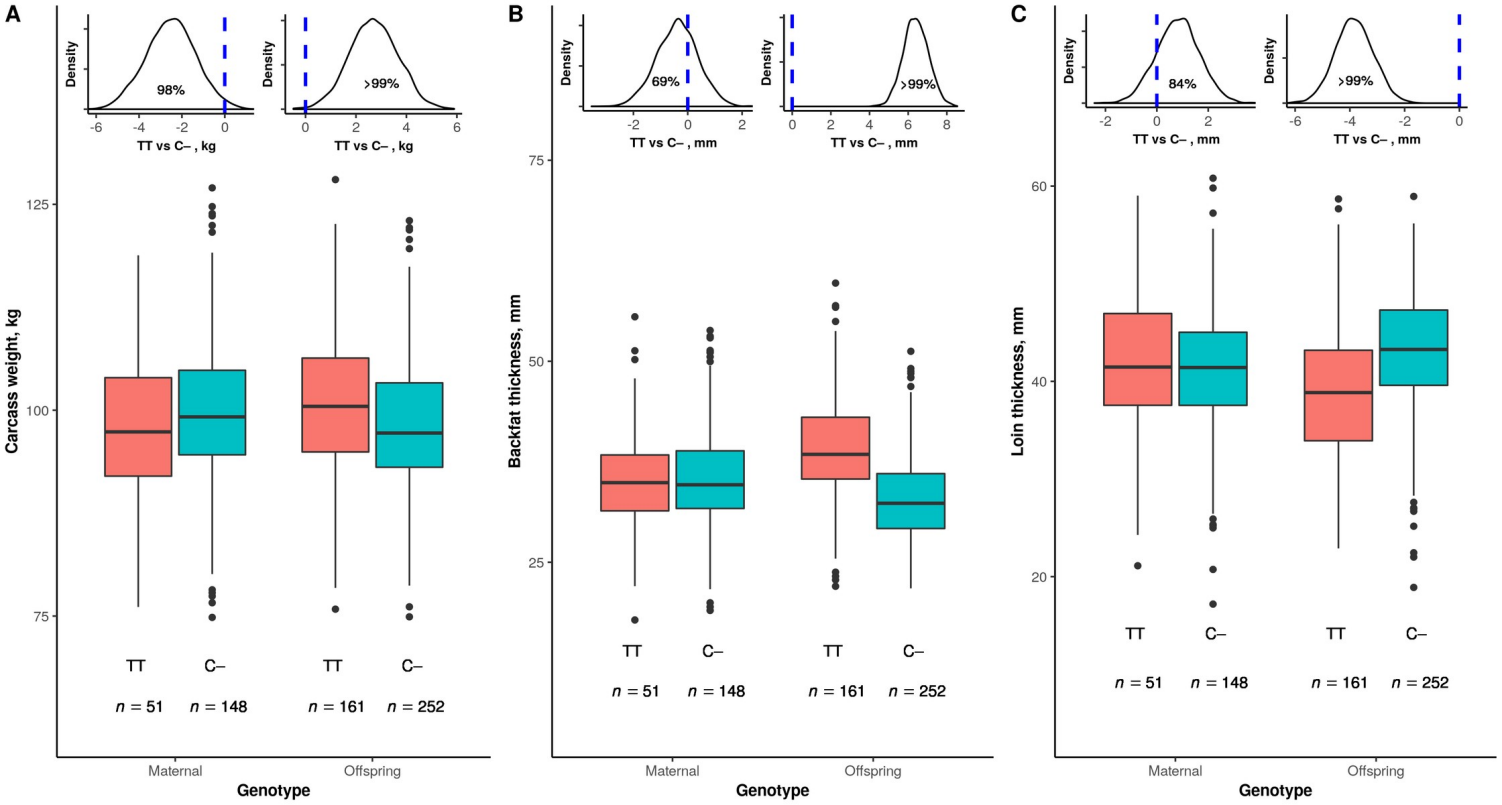
Antagonistic maternal and direct effect of *LEPR* gene on body weight at the end of the growing period. Boxplot distribution of carcass body weight (*A*), backfat thickness (*B*), and loin thickness (*C*) at 223 days of age by maternal (sow) and offspring (pig) *LEPR* genotype. Values represented are adjusted for systematic effects. For each trait, the marginal posterior distribution of the difference between TT and C–genotypes is depicted on the top of each panel, with the blue dotted line indicating the zero value (no difference) and the accompanying percentage standing for the posterior probability of TT being higher (area under of the curve at the right side of the line) or lower (area under of the curve at the left side of the line) than C–. *LEPR* genotypes were considered to differ if this probability value was ≥95%. Sample size (*n*) is given below each boxplot.

In a first experiment, we proved that the *LEPR* gene is a source of maternal effects. We measured the carcass weight of 413 pigs from 199 sows and 18 sires that were individually traced from birth to slaughter. In line with previous results in this Duroc line [20], we grouped the CC and CT genotypes in a single class (C−) due to the recessive nature of the T allele, which was also evidenced in the set of pigs used here, particularly for carcass weight (S1 Table). The TT sows exerted a negative impact on the carcass weight of their offspring at the end of the growing period of similar extent to the positive direct effect of the TT genotype over each individual (Fig 1*A*). Thus, TT pigs from TT dams (*n* = 62; mean = 97.7 kg) were about as heavy as C–pigs from C–dams (*n* = 207; mean = 97.6 kg; difference: +0.1 kg; posterior probability of the difference being greater than zero: P(>0) = 0.53), but TT pigs from C–dams (*n* = 99; mean = 100.5 kg) were around 5% heavier (+5.2 kg, P(>0) >0.99) than C–pigs from TT dams (*n* = 45; mean = 95.3 kg). In contrast, body composition was only influenced by *LEPR* direct effects, with TT pigs gaining more fat (Fig 1*B*) and less lean (Fig 1*C*) than C–pigs. Data collected in the next series of experiments enabled us to show that the thriftier behavior of the TT genotype is behind the antagonism between direct and maternal effects for body weight caused by the *LEPR* gene.

### Maternal influence

**Fig 2:**
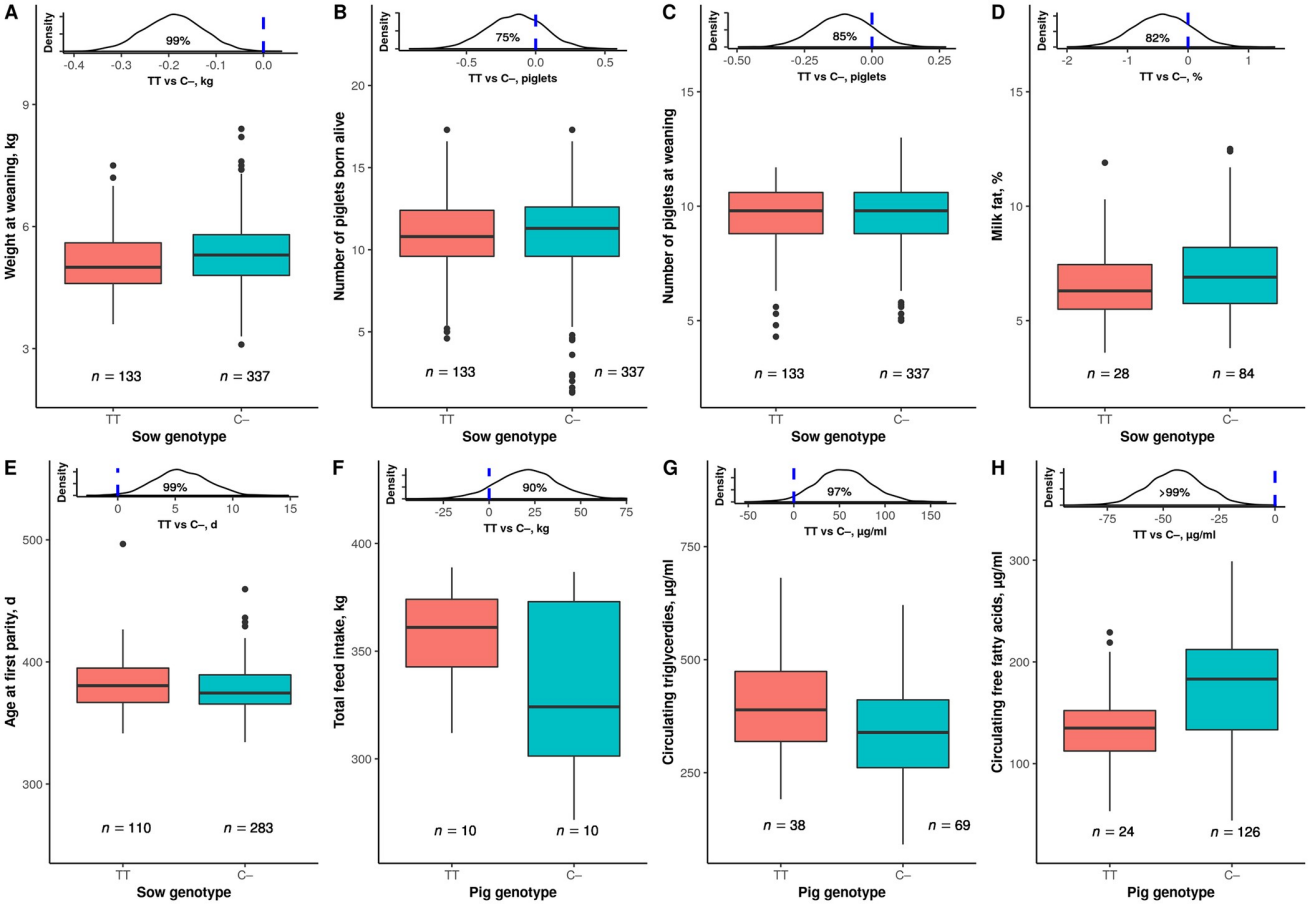
Effects of *LEPR* genotype on maternal and individual body energy traits. Boxplot distribution by the sow *LEPR* genotype for the average weight of piglets at weaning (*A*), number of piglets born alive (*B*) and at weaning (*C*) per litter, fat content in milk at day 6 of lactation (*D*), and age at first parity (*E*), as well as for total feed intake from 70 to 200 days of age (*F*) and blood circulating concentration of triglycerides after a 12-h fast (*G*) and total free fatty acids after a 24-h fast (*H*) in pigs of 184 and 175 days of age, respectively. Data displayed as in Fig 1.

Energy intake that exceeds expenditure is the driver of weight gain. Until weaning, the limiting maternal resource for piglet growth is the sow milk production [22, 23]. In a second experiment, which involved 927 weaned litters from TT (*n* = 133) and C–(*n* = 337) sows, we demonstrated that the piglets born from TT sows were lighter at weaning than those born from C–sows (-150 g, posterior probability of the difference being lower than zero: P(<0) >0.99; mean weight of piglets from C− sows = 5.3 kg; Fig 2*A*), which indirectly implies less milk production in TT sows than in C− sows. This decline could not be ascribed to differences in litter size, either at birth (Fig 2*B*) or at weaning (Fig 2*C*), nor to different parity number. Primiparous sows, which produce less milk [24], were evenly distributed across genotypes (51.7% and 47.8% of total litters from TT and C–sows, respectively) and weights were adjusted for parity number. Moreover, piglets from primiparous TT sows were also lighter at weaning compared with those from primiparous C–sows (–132 g, P(<0) >0.99; mean weight of piglets from C− sows = 4.9 kg). The detrimental effect of TT sows on litter weight was likely underestimated, given that TT piglets, which are expected to grow more rapidly, were overrepresented in litters from TT sows compared to C–sows. In line with commercial practice, litter size was equalized by cross-fostering within 24 h of birth and creep feed was offered to all litters from 10 days after birth until weaning. Although genotype was not considered for adoptions, solely 31.5% of the litters received piglets (on average 2.4 piglets) from other litters and, therefore, cross-fostering only partially broke down the correlation between parent and offspring genotypes. On the other hand, creep feed has no effect on growth of early-weaned piglets [25, 26]. Most pigs are non-consumers and, besides, consumers are in fact lighter piglets [27]. We did not find enough evidence that TT sows had lower milk quality in terms of fat content (Fig 2*D*) and fatty acid composition (S2 Table). The negative maternal effect of TT sows on piglet growth arises from saving extra energy at the expense of reproduction. The later age at first parity of TT sows reinforced this assertion (*+*5.5 d, P(>0) = 0.99; mean of C–sows = 377.3 d; Fig 2*E*).

### Individual influence

This thrifty behavior should be also expected for TT growing pigs. In a last series of experiments, we verified that TT growing pigs showed a more positive energy balance than C–pigs. They were not only more prone to eat more (+20.9 kg of feed, P(>0) = 0.90; mean of C–pigs = 333.4 kg; Fig 2*F*), as already observed in Duroc x Iberian crossbreds [28], but they also burnt off fewer calories. In two *ad hoc* trials we proved that, after fasting, TT pigs exhibited higher circulating levels of triglycerides (*+*57 μg/mL, P(>0) = 0.97; mean of C–pigs = 347 μg/mL; Fig 2*G*) and lower levels of total free fatty acids (–43 μg/mL, P(<0) >0.99; mean of C–pigs = 179 μg/mL; Fig 2*H*) compared to C–pigs. Even in energy-demanding scenarios, the metabolism of TT pigs favored fatty acid uptake over release.

## Discussion

Direct and maternal sources of variation are usually examined using biometrical models [29, 30]. The model predominantly used postulates that the observed phenotype of an individual is the sum of a phenotypic direct effect due to the individual itself and a phenotypic maternal effect due to its dam [30, 31]. Direct and maternal phenotypes are accommodated into the model as the sum of additive genetic and environmental effects, which are individually fitted making use of available pedigree relationships [32, 33]. The implementation of this model is highly demanding, requiring sizeable datasets including dam-offspring pairs with records [34]. This population structure is difficult to obtain even in large commercial animal populations and thus, for simplicity, maternal effects have mostly been estimated assuming that dam and offspring are not environmentally correlated. Because this may lead to biased and inaccurate estimates, reported estimates of the genetic correlation between direct and maternal effects (often strongly negative) have been questioned, if not considered statistical artifacts [7, 31]. Despite the momentum of genomic discovery, there is still a lack of genetic support for a negative causal link between direct and maternal effects. In this study, using the *LEPR* gene as an example, we provide evidence of genes that simultaneously influence direct and maternal effects with opposite effects on body growth. Maternal effects may appear as a result of maternal imprinting [35], but this is inconsistent with the recessive inheritance of *LEPR* [19, 20] and with findings that point to paternal rather than maternal expression in the region around *LEPR* [14]. Furthermore, results on *LEPR* in particular allow drawing biological and evolutionary consequences.

Many environmental factors affect pre-weaning growth, such as maternal nutrition and feeding regime [36]. Diets for accelerated prepubertal growth rate decrease subsequent milk production as a result of impaired mammary development due to the higher energy demand for growth [37, 38]. In contrast, a high feed intake during gestation is beneficial for offspring performance [36]. However, this only applies if the sow is able to act as an energy buffer. Thus, heavier and fatter sows at parturition only produce heavier piglets at weaning as long as they are able to mobilize during lactation the energy surplus that they accumulated during gestation [11]. During lactation, the adipose tissue shifts towards greater net rates of lipolysis, thus elevating the concentration of circulating free fatty acids and glycerol in blood for use as energy substrates [39]. Furthermore, increased adipose tissue results in greater release and lower clearance of free fatty acids [40]. As a signal of available energy, leptin is expected to enhance lipolytic activity [41] and investment into reproduction [42]. However, TT pigs present the opposite pattern despite their increased levels of leptin relative to C–pigs [20], with less free fatty acids available in blood and impaired maternal environment. In line with individuals with hyperleptinemia due to defective leptin receptor signaling, the T allele attenuates but not inactivates [13] the function of leptin as a mediator between self-maintenance and offspring investment [42]. In this trade-off, the TT sows tip the balance towards self-maintenance, and by doing so, they indirectly outsource part of their reproductive success to the offspring. The absence of an antagonistic maternal effect for fat and lean mass would explain why direct effects are more easily detected in body composition [19, 20] than in growth traits, for which direct effects only become entirely apparent in pigs raised by sows with genotype other than theirs. Similarly, direct effects for body weight stand out at advanced ages as adipose tissue develops. Unlike nutrition-induced changes, the effects of *LEPR* are genetics-driven and, as such, have implications that extend over generations.

Growth and energy balance involve a set of traits potentially subjected to selection response and evolutionary change. As causal of both direct and maternal effects, *LEPR* benefits reproductive success by providing a system that allocates resources to the sow or the offspring according to whether selective pressure is stronger before or after weaning. In this way, *LEPR* contributes to uplift the population carrying capacity for a given environment. The fact that the T allele is fixed in the Iberian breed, which has traditionally been reared outdoors under limited and fluctuant feed resources [43], and present at a relative high frequency in the Duroc lines wherein selective breeding favored heavy and fat pigs [44] (like Duroc-line 1 in S3 Table), can be interpreted as proof-of-principle of the plasticity of the system. Weaning covers the transition period where piglets switch from feeding on sow milk to solid feed. In natural conditions, this is a gradual process that may last until up to 22 weeks of age [45]. Feed restrictions during this time prompt piglets to seek out food earlier to compensate for a lower energy intake from milk [26]. In this setting, the T allele, with a favorable direct effect for growth and fat accumulation, has a greater chance of increasing to a high frequency. Contrarily, in most commercial lines, where pigs are selected for lean efficiency under high feeding and management standards, the T allele, associated with decreased capacity for maternal ability and leanness, is no longer beneficial and therefore tends to be swept away (S3 Table). Similar mutations may exist in other pig breeds and species. Significantly, the *LEPR* promoter region shows great haplotypic diversity in wild boar [46], which can persist in very diverse habitats. Evidences of interaction between maternal and direct *LEPR* effects for body weight and fat mass have also been reported in humans [47]. The *LEPR* model provides a sensible biological mechanism for transgenerational energy allocation that could be extended with other genes.

## Material and methods

### Animals, records and samples

All experimental pigs were from a purebred Duroc line mainly selected for an index including lean growth and intramuscular fat content [48] and primarily used for producing high-quality dry-cured products. Five independent experiments were conducted to examine maternal and individual influences (S4 Table). In the first experiment, twelve batches of barrows (n = 413) were raised under standard commercial conditions. At about 10 weeks of age pigs were moved to the fattening units, where they were allocated by sex in pens of 8 to 12 individuals and were given ad libitum access to commercial diets. Pigs raised at the same time and in the same farm were considered as one batch. All batches were slaughtered in the same abattoir at around 32 weeks of age (223 days, 11 SD), where carcass weight was recorded, and carcass backfat and loin thickness were ultrasonically measured with an automatic carcass grading equipment (AutoFOM, SFK-Technology, Denmark) at 6 cm off the midline between the third and fourth last ribs. Data used in the second experiment were obtained from a sow farm managed using standard practices, where gilts were monitored for oestrus at 6.5 months of age and then bred on their second detected oestrus. Sows were rebred on their first oestrus after weaning. Sows were rebred on their first oestrus after weaning. The sow (n = 430) performance by parity including age at parity, number of piglets born alive, number of weaned piglets and litter weight at weaning (23 days, 2 SD) was recorded for 26 months (10 contemporary year-season farrowing batches). Creep feed was offered to litters from about 10 days after birth until weaning. In a random set of primiparous sows (n = 112), a 15-mL sample of milk was extracted at around the end of the first week of lactation (6 days, 3 SD) from anterior teats following intramuscular oxytocin injection (20 UI; Hormonipra, Spain). Milk samples were stored at -40°C until analysis. In the third, fourth and fifth experiments, five additional batches of barrows, identically raised as in the first experiment, were used to examine feed intake (one batch, n = 20) and circulating triglycerides (two batches, n = 107) and free fatty acids (two batches, n = 150). In the first batch, feed intake from 70 days (1 SD) to 200 days (1 SD) was individually monitored in 10 full- or half-sibs pairs of different *LEPR* genotype (TT and C−) using an automatic feeding system (IVOG^®^, Insentec, Netherlands). Sib-pairs were allocated in pens with other 10 individuals. In the other four batches, blood samples were collected using 8.5-mL serum (BD Vacutainer^®^ SST^™^ II Advance, Franklin Lakes, NJ, USA) or plasma tubes (BD Vacutainer^®^ K2-EDTA, Franklin Lakes, NJ, USA) by jugular venipuncture at 184 days of age (4 SD) after 12-h fasting (for serum triglycerides) or at 175 days of age (6 SD) after 24-h (for plasma free fatty acids) fasting and were centrifuged (3,000 × g for 10 min at 4 °C). Harvested serum and plasma samples were stored at 4 °C and –80 °C, respectively, for subsequent analysis. Finally, we used genomic DNA from commercial genetic types and European wild boar specimens for monitoring allele segregation. All pigs used in the study were raised and slaughtered in commercial units following applicable regulations and good practice guidelines on the protection of animals kept for farming purposes, during transport and slaughter. The specific protocols for batches in the third series of experiments were approved by the Ethical Committee on Animal Experimentation of the University of Lleida (CEEA 08/01–12 and CEEA 05-04/15).

### Genotyping

All sows and pigs used in the experiments were genotyped for *LEPR* (rs709596309; C>T; on SSC6) single nucleotide polymorphism. Genomic DNA was isolated from biological samples using a standard protocol. Quantification and purity of DNA was assessed by spectrophotometry with a NanoDrop N-1000 Spectrophotometer (Thermo Fisher Scientific, Waltham, MA, USA) and the integrity was tested by electrophoresis in agarose gels. The *LEPR* polymorphism was genotyped by real time qPCR (QuantStudio3, Applied Biosystems, Waltham, MA, USA) with High Resolution Melt analysis. Primers used for genotyping the region containing the target [19] are: forward 5’-CAGAGGACCTGAATTTTGGAG and reverse 5’-CATAAAAATCAGAAATACCTTCCAG. The PCR reaction was performed in a final volume of 5 μl including 1x Thermo Scientific^™^ Luminaris Color HRM qPCR Master Mix (Thermo Fisher Scientific, Waltham, MA, USA), 0.4 μM of each primer, and 20 ng of genomic DNA. Thermocycling conditions were 50 °C 2 min, 95 °C 10 min, and 40 cycles of 95 °C 15 sec, 60 °C 1 min, followed by a high-resolution melting curve starting with a denaturation at 95 °C for 15 sec, annealing at 60 °C for 1 min and a slow ramp at 0.015 °C/sec up to 95 °C. High Resolution Melt software v3.1 (Applied Biosystems, Thermo Fisher Scientific, Waltham, MA, USA) was used for the melting data analysis and the genotyping of the samples.

### Milk analysis

Milk fat content and fatty acid composition were determined in duplicate using the gravimetric solvent method of Hara and Radin [49] as adapted by Feng et al. [50] followed by gas chromatography [51]. Determinations were performed in duplicate. Milk samples were homogenized in a shaking water bath at 37 °C, 100 rpm for 5 min, and 500-μl aliquots were extracted. A solution of hexane:isopropanol (3:2 vol/vol) was added into each aliquot and the mixture was stirred for 30 min at room temperature. Then, 2 mL of sodium sulfate solution (12%) were added in the mixture to separate hexane from isopropanol by centrifugation (5 min 3000 rpm) and the upper hexane layer was transferred to a 15 mL tube. The mixture was washed again with 2 mL hexane to recover the remaining lipid fraction and then placed into a rotary evaporator to remove any exceeding hexane (for 30 min at 40 °C). The lipid fraction was dried with nitrogen until constant weight to determine fat content. To determine fatty acid composition, the lipid content was resuspended using a solution of boron trifluoride 20% in methanol to obtain fatty acid methyl esters by transesterification. Fatty acid methyl esters were analysed by gas chromatography with a capillary column DB-23 PN (30 m x 0.25 mm, Agilent Technologies, Santa Clara, C, USA) and a flame ionization detector with helium as the carrier gas at 1 mL/min. The quantification was carried out through area normalization using tripentadecanoin (C15:0) as an internal standard. The amount of each fatty acid was expressed as the percentage of each individual fatty acid relative to total fatty acid (S2 Table).

### Triglycerides and free fatty acid quantification

Serum triglyceride levels were measured enzymatically using a commercial kit (GPO-PAP colorimetric enzyme test, Olympus diagnostics, Clare, Ireland). Plasma free fatty acids were extracted following the method described by Hellmuth et al. [52] and quantified by the multiple reaction monitoring (MRM) approach using an ultra-high performance liquid chromatography (UHPLC) on an Acquity UPLC, HSS T3 column (2.1 × 150 mm; 1.8 μm particle size) coupled to a Xevo TQ-S mass spectrometer (Waters, Milford, MA, USA). Data were processed using QuanLynx^®^ software, with palmitic fatty acid-d_31_ as internal standard. Total free fatty acid content in plasma was calculated as the sum of individual fatty acids the content of which was at least 0.4 μg/mL [53].

### Models and distributions

The direct (pig) and maternal (sow) effects for carcass traits (weight, backfat thickness and loin thickness) due to the *LEPR* genotype were estimated independently for each trait. In matrix notation, the animal model was **y** = **Xb** + **Za** + **e**, where **y** is the vector of observations for a trait; **b**, **a** and **e** are the vectors of systematic (pig and sow *LEPR* genotype, batch and age at measurement as a covariate), polygenic and residual effects, respectively; and **X** and **Z** are the incidence matrices that relate **b** and **a** with **y**, respectively. The haplotype additive (a) and dominant (d) effects were tested replacing the genotype effect by the covariates a (TT: 1, CT: 0, CC: -1) and d (TT: 0, CT: 1, CC: 0). The traits were assumed to be conditionally normally distributed as [**y** | **b**, **a**, **I**σ_e_^2^] ~ *N* (**Xb** + **Za**, **I**σ_e_^2^), where σ_e_^2^ is the residual variance and **I** the appropriate identity matrix. The animal effects conditional on the additive genetic variance σ_a_^2^ were assumed multivariate normally distributed with mean zero and variance **A**σ_a_^2^, where **A** was the numerator relationship matrix calculated from a two-generation pedigree. Other traits measured only once, either in the pig (feed intake, serum triglycerides and plasma free fatty acids) or in the sow (age at first parity and milk fat content), were analyzed with the same model but only including the genotype of the pig or the sow where applicable. Pigs in a given batch were contemporaneous raised and tested at the same time. Sow records with repeated measurements (number of piglets born alive, number of weaned piglets and litter weight at weaning) were analyzed with a repeatability model that accounts for the polygenic effect of the sow and with the sow *LEPR* genotype, the parity number (from 1 to 6) and the batch as systematic effects. Litters in a given batch were born in the same year and season. The same distributions as above were assumed.

### Inference

Statistical inferences for each of the above models were derived from the samples of the marginal posterior distribution using a Gibbs sampling Markov chain Monte Carlo algorithm with a chain of 500,000 iterations, where the first 100,000 were discarded and one sample out of 100 iterations retained. Software and source code is available (Legarra et al., 2008; http://genoweb.toulouse.inra.fr/~alegarra/tm_folder [deposited: 3 August 2011]). Flat priors were used for **b**. Convergence was tested using the Z-criterion of Geweke (1992) and visual inspection of convergence plots. Statistical evidence for the direct and maternal effects of the *LEPR* polymorphism was calculated as the marginal posterior probability of the difference between genotype estimates being greater or lower than zero. We considered that there was strong (suggestive) evidence of difference between the genotypes when the probability of that difference being greater or lower than zero was of at least 95% (90%).

## Supporting information

S1 Table Difference between *LEPR* (rs709596309 C>T) genotypes and additive and dominant effects for investigated traits. (PDF)

S2 Table Effects of *LEPR* (rs709596309) genotype on sow milk fatty acid composition. (PDF)

S3 Table Frequency of *LEPR* (rs709596309; C>T) genotypes and gene frequency of the allele T (q) in different pig breeds and crosses. (PDF)

S4 Table Summary of experimental data. (PDF)
