# Supplementary material for: Antagonistic maternal and direct effects of the leptin receptor gene on body weight in pigs
Source: PLoS One. 2021 Jan 28;16(1):e0246198. doi: 10.1371/journal.pone.0246198 (PMC7842917; doi:10.1371/journal.pone.0246198)
Supplement: S1 Table — (PDF) [file pone.0246198.s001.pdf]

S1 Table. Difference between *LEPR* (rs709596309 C>T) genotypes and additive and dominant effects for investigated traits. Mean of the estimated marginal posterior distribution of the difference between *LEPR*-TT and *LEPR*-CC genotypes with respect to the *LEPR*-CT genotype (TT-CT and CC-TT, respectively) and additive (a) and dominant (d) values for the *LEPR*-T allele (in parentheses, the probability of each value being greater than zero) for direct and maternal effects.

**Trait**	**Genotype difference**		**Genetic effects**	
	**TT − CT**	**CC − CT**	**a**	**d**
**Direct effects**				
Carcass weight, kg	3.0 (**1.00**)	0.2 (0.57)	1.9 (**1.00**)	-1.9 (**0.01**)
Backfat thickness, mm	6.0 (**1.00**)	-1.8 (**0.04**)	4.6 (**1.00**)	-4.1 (**0.00**)
Loin thickness, mm	-3.6 (**0.00**)	1.2 (0.87)	-2.8 (**0.00**)	2.4 (**1.00**)
Weight at weaning, g	-180 (**0.00**)	20 (0.59)	-110 (**0.00**)	100 (**0.96**)
No. of piglets born alive	-0.2 (0.23)	-0.1 (0.40)	-0.1 (0.31)	0.1 (0.73)
No. of piglets at weaning	-0.1 (0.26)	0.1 (0.66)	-0.1 (0.18)	0.02 (0.60)
Age at first parity, days	5.8 (**0.99**)	1.6 (0.71)	2.5 (**0.94**)	-4.1 (**0.02**)
Milk fat, %	-0.2 (0.33)	0.2 (0.62)	-0.2 (0.28)	0.1 (0.56)
Plasma triglycerides, µg/ml	53.3 (**0.95**)	-0.9 (0.49)	3.0 (**0.94**)	-3.2 (0.14)
Plasma free fatty acids, µg/ml	-53.6 (**0.00**)	-12.8 (0.11)	-13.0 (**0.02**)	21.2 (**0.98**)
**Maternal effects**				
Carcass weight, kg	-2.3 (**0.04**)	1.6 (0.85)	-2.0 (**0.01**)	0.8 (0.78)
Backfat thickness, mm	-0.8 (0.18)	-0.9 (0.20)	-0.1 (0.44)	0.5 (0.74)
Loin thickness, mm	1.0 (0.88)	0.6 (070)	0.4 (0.72)	-0.6 (0.21)
