# Supplementary material for: Antagonistic maternal and direct effects of the leptin receptor gene on body weight in pigs
Source: PLoS One. 2021 Jan 28;16(1):e0246198. doi: 10.1371/journal.pone.0246198 (PMC7842917; doi:10.1371/journal.pone.0246198)
Supplement: S2 Table — (PDF) [file pone.0246198.s002.pdf]

S2 Table. Effects of *LEPR* (rs709596309) genotype on sow milk fatty acid composition. Mean of the estimated marginal posterior distribution of the difference between sow *LEPR* genotypes (TT, *n*=28, and C−, *n*=84) and probability of this difference being greater than zero (P>0) for fatty acid composition (% of total fatty acids) of sow milk. *LEPR* genotypes were considered to differ if this probability value was <0.05 (C− is higher than TT) or >0.95 (TT is higher than C−).

**Fatty acid, %**	**Mean**	**SD**	**Difference TT − C−**	**P>0**
Capric acid (C10:0)	0.2	0.1	-0.03	0.12
Lauric acid (C12:0)	0.4	0.3	-0.03	0.27
Myristic acid (C14:0)	3.5	0.9	0.13	0.78
Myristoleic acid (C14:1n5)	0.2	0.1	-0.02	0.21
Palmitic acid (C16:0)	28.1	3.5	0.87	0.92
Palmitoleic acid (C16:1n7)	8.7	2.7	0.36	0.76
Stearic acid (C18:0)	4.2	0.8	-0.18	0.13
Vaccenic acid (C18:1n7)	2.5	0.5	-0.17	0.01
Oleic acid (C18:1n9)	33.9	5.6	-0.52	0.32
Linoleic acid (C18:2n6)	14.7	3.7	-0.48	0.23
α-linolenic acid (C18:3n3)	1.1	0.3	0.01	0.54
Eicosanoid acid (C20:1n9)	0.4	0.2	<0.01	0.49
Eicosadienoic acid (C20:2n6)	1.0	0.5	-0.06	0.20
Saturated fatty acids	36.6	4.0	0.75	0.85
Monounsaturated fatty acids	46.1	4.7	-0.32	0.36
Polyunsaturated fatty acids	17.3	3.4	-0.45	0.25
