# Supplementary material for: Antagonistic maternal and direct effects of the leptin receptor gene on body weight in pigs
Source: PLoS One. 2021 Jan 28;16(1):e0246198. doi: 10.1371/journal.pone.0246198 (PMC7842917; doi:10.1371/journal.pone.0246198)
Supplement: S3 Table — (PDF) [file pone.0246198.s003.pdf]

S3 Table. Frequency of *LEPR* (rs709596309; C>T) genotypes and gene frequency of the allele T (q) in different pig breeds and crosses.

		LEPR genotype			
**Genetic type**	**No. of pigs**	**CC**	**CT**	**TT**	**q**
Iberian	92	0	0	92	1.00
Duroc (line 1)	1135	345	539	251	0.46
Duroc (line 2)	25	13	10	2	0.28
Landrace	33	32	0	1	0.03
Pietrain	29	17	11	1	0.22
Landrace x Large White	142	122	20	0	0.07
Landrace x Duroc	12	9	3	0	0.12
Wild boar	7	7	0	0	0.00
