# Supplementary material for: Antagonistic maternal and direct effects of the leptin receptor gene on body weight in pigs
Source: PLoS One. 2021 Jan 28;16(1):e0246198. doi: 10.1371/journal.pone.0246198 (PMC7842917; doi:10.1371/journal.pone.0246198)
Supplement: S4 Table — (PDF) [file pone.0246198.s004.pdf]

S4 Table. Summary of experimental data.

Experiment	Trait	No. of batches	No. of records by *LEPR* genotype		Mean	SD
			TT	C–		
**1**	Carcass weight, kg	12	161	252	99.4	9.9
	Backfat thickness, mm	12	161	252	35.4	7.9
	Loin thickness, mm	12	161	252	41.1	7.1
**2**	No. of sows	10	133	337	-	-
	No. of parities	10	248	679	-	-
	Weight at weaning, kg	10	248	679	5.2	0.9
	No. of piglets born alive	10	248	679	11.0	2.4
	No. of piglets at weaning	10	248	679	9.7	1.3
	Age at first parity, days	8	110	283	378.8	22.1
	Milk fat, %	3	28	84	7.0	2.8
**3**	Total feed intake, kg	1	10	10	345.5	35.8
**4**	Plasma triglycerides, µg/ml	2	38	69	367.0	139.5
**5**	Plasma free fatty acids, µg/ml	2	24	126	174.9	58.2
